# The Etiological Profile of Adrenal Incidentalomas

**DOI:** 10.7759/cureus.32564

**Published:** 2022-12-15

**Authors:** Fatima-Zahra Lahmamssi, Loubna Saadaoui, Hayat Aynaou, Houda Salhi, Hanan El Ouahabi

**Affiliations:** 1 Endocrinology, Diabetology, Metabolic Diseases and Nutrition, Hassan II University Hospital, FES, MAR; 2 Laboratory of Epidemiology and Research in Health Sciences, Faculty of Medicine and Pharmacy, Sidi Mohamed Ben Abdellah University, FES, MAR

**Keywords:** adrenalectomy, washout, etiology, cortisolic adenoma, adrenal incidentalomas

## Abstract

Introduction

An adrenal incidentaloma (AI) is an unsuspected tumor in one or both adrenal glands, which is discovered incidentally on an imaging exam not prompted by adrenal exploration. The etiologies can be multiple; they condition therapeutic management. The objective of our study is to describe the etiological and therapeutic profiles of AI in our department.

Materials and methods

A retrospective study was carried out in the Endocrinology, Diabetology, and Nutrition Department of the Hassan II University Hospital of Fez on patients managed for AI from September 2009 until March 2022. We included all the patients who were followed and/or hospitalized for adrenal incidentalomas.

Results

There were 86, predominantly female, patients (67.85%). The mean age was 58.91+/-14.40 years. The clinical findings were a unilateral adrenal mass in 73.25% of patients, localized on the left in 39.53%, on the right in 33.72%, and a bilateral one in 26.75%. Its size varied from 12 to 196 mm, with an average of 35.5 mm. The most common etiologies found in our series were a non-functional adrenal adenoma in 54.56%, a subclinical cortisolic adenoma in 19.76%, an adrenocortical carcinoma in 5.81%, and a pheochromocytoma in 5.81%. Adrenalectomy was indicated in 19.76% of our patients, 17.44% were monitored closely, 20.94% were monitored for comorbidities, and 41.86% had been advised to abstain from treatment.

Conclusion

An adrenal incidentaloma has become more and more frequent. It constitutes an entity with various etiologies, which can be serious. The main etiology in our series was non-functioning adrenal adenoma, for which therapeutic abstention was indicated in 48% of cases.

## Introduction

Adrenal incidentalomas (AIs) are commonly discovered incidentally on cross-sectional abdominal imaging (CT, MRI, and rarely ultrasound) performed for reasons other than adrenal mass. These incidentalomas can be hormonally functional or not and benign or malignant. In the majority of cases. The clinical manifestations vary according to the etiology. The most common etiologies are non-secreting adenomas, subclinical cortisolic adenomas, pheochromocytoma, and rarely adrenal carcinomas or metastases. In order to determine the risk of malignancy of the adrenal mass, a CT scan with and without injection of iodinated contrast is the gold standard [1].

The modalities of management and monitoring depend on the nature of the lesion (benign or malignant), its hormonal status, and its symptomatologic impact [1]. Management may be surgical or based on close monitoring that may redirect to surgery if there is a change in size or signs of hormonal hypersecretion. The objective of our study is to describe the clinical, paraclinical, etiological, and therapeutic profiles of adrenal incidentalomas in our department.

## Materials and methods

Study design and setting

A retrospective, descriptive, and analytic study was conducted over a period of 13 years from September 2009 to March 2022. The study concerned 112 patients who were hospitalized for exploration of an adrenal incidentaloma and followed in the Endocrinology Diabetology and Nutrition Department of the Hassan II Hospital in Fez.

Eighty-six patients who were followed for an adrenal incidentaloma and had complete medical records on the different clinical, paraclinical, therapeutic, anatomopathological, and evolutionary aspects were included. On the other hand, 26 patients were excluded, including 19 patients lost to follow-up and 7 patients with missing data on the anatomopathological study and evolutionary data.

Data collection and assessment

The following data were collected from the computerized medical records of patients with AI and were reported on a field worksheet and then integrated into Microsoft Excel (Microsoft Corporation, Redmond, WA). The sociodemographic variables were age, gender, and geographical origin. Clinical data were a history of diabetes, high blood pressure, tuberculosis infection, a neoplastic pathology, abdominal trauma, and contact with dogs. The paraclinical variables were biological and consisted in a hormonal assessment and radiological, which contained an abdominal ultrasound, scanner, and magnetic resonance imaging. From these clinical and paraclinical data, we were able to establish an etiological diagnosis, and patients were classified according to the following therapeutic modalities: surgical treatment, abstention, or surveillance. Finally, we collected the anatomopathological reports of the operated patients and their evolution.

Statistical analysis

The results are expressed either with the mean ± standard deviation or in the form of medians or percentages for the numerical variables and with the numbers and percentages for the qualitative variables. The coding and processing of the collected data were done using Excel software, and the statistical analysis was done using Statistical Package for the Social Sciences (SPSS) version 18 for Windows SPSS Inc. Released 2009. PASW Statistics for Windows, Version 18.0. Chicago: SPSS Inc.

Ethics statement

Anonymity and confidentiality were maintained for all participants.

## Results

A total of 86 subjects were included in the study; they were diagnosed fortuitously with adrenal incidentalomas. The mean age of our patients was 58.91+/-14.40 years, with a female predominance (67.85%). The main functional signs reported by our patients were digestive; the rest of the symptoms are listed in Table 1. 

**Table 1:** Functional signs reported in our series This table summarizes the different functional signs perceived by the patients in our series.

Functional signs	Number of cases	Percentage %
Digestive disorders: Abdominal pain, low back pain	45	52.32%
Menard's triad (headache, palpitation, sweating)	2	2.32%
Subclinical Cushing's syndrome	12	13.95%
Altered general condition (asthenia, anorexia, weight loss)	6	6.97%
Neurosensory signs of hypertension	20	23.25%
Signs of hypokalemia	1	1.16%

Physical examination of our patients showed arterial hypertension in 43%, abdominal tenderness in 33.3%, and normal in 21.4%. The rest of the physical signs are grouped in Table 2.

**Table 2:** Main physical signs reported in our series Obesity and abdominal sensibility were the most predominant signs in our series.

Physical signs	Number of cases	Percentage %
Hypertension	37	43%
Obesity	28	33.33%
Abdominal sensibility	28	33.33%
Abdominal mass	0	0
Subclinical Cushing's syndrome	12	14.28%
Signs of hyperandrogenism	2	2.3 %
Goiter	20	23.8%
Splenomegaly	2	2.3%
Hepatomegaly	1	1.1%
History of mastectomy	1	1.1%
Normal	18	21.42%

In our series, a unilateral mass was found on imaging in 73.25% of patients, located on the left in 39.53%, on the right in 33.72%, and bilateral in 26.75%. Its size varied from 12 to 196 mm, with an average of 35.5 mm, and in the case of bilateral adrenal incidentalomas, the size of the largest tumor was taken into account. 

Among these masses found on imaging, 15.11% were suspected of malignancy, showing an adrenocortical carcinoma in 5.81% of cases with central necrosis and invasion of the surrounding organs. Pheochromocytoma was also found in 5.81%, adrenal metastases in 2.32%, and malignant Hodgkin's lymphoma in 1.1% of cases.

The hormonal exploration of our patients showed in 5.8% of cases an elevation of urinary metanephrine with a mean of 775±536 ug/24h (reference values <31), an elevation of urinary normetanephrine with a mean of 612±265 ug/24h (reference values <51), and an elevation of urinary 3 orthomethyl dopamine with a mean of 420±130 ug/24h (reference values <55). An elevated plasma cortisol level after a minute braking test with 1 mg of dexamethasone in 19.7% of the cases with values between 1.9 and 5 ug/dl (which corresponds to possible autonomous cortisol secretion) in 13.95% and above 5 ug/dl (which corresponds to an autonomous cortisol secretion) in 5.81%. Adrenocorticotropic hormone (ACTH) was measured preoperatively in 6.9% of patients with subclinical hypercortisolism to determine the degree of suppression of the corticotropic axis and whether these patients will require a relay with hydrocortisone postoperatively. Plasma aldosterone and renin with the ratio of aldosterone to renin were measured in patients with adrenal incidentaloma with hypertension +/- hypokalemia and were positive in only one case.

After hormonal exploration, etiological diagnoses were made, which were dominated by non-functioning adrenal adenomas in 54.56% of cases. The other etiologies are presented in Table 3.

**Table 3:** The main etiologies found in our series Non-functional adrenal adenomas and subclinical cortisolic adenomas were the most predominant etiologies in our series.

Adrenal tumors	Number of cases (86)	Percentage %
Non-functional adrenal adenomas	47	54.56%
Pheochromocytoma	5	5.81%
Subclinical cortisolic adenoma	17	19.76%
Adrenocortical carcinoma	5	5.81%
Adrenocortical hematoma	3	3.48%
adrenal metastases	2	2.32%
Adrenal cyst	2	2.32%
Myelolipoma	3	3.48%
Primary Surrenal Lymphoma	1	1.16%
Adenoma of conn (primary hyperaldosteronism)	1	1.16%

Concerning the management, adrenalectomy was indicated in 19.76% of our patients, 17.44% were monitored closely, 20.94% had been monitored for comorbidities, and 41.86% had been advised to abstain from treatment.

Pheochromocytoma was histologically confirmed in 5.8% of cases with a mean tumor size of 7.6+/-2.8 cm. Pheochromocytoma of the Adrenal gland Scaled Score (PASS) score <3 (which is in favor of a tumor of non-aggressive potential) in 3.4% and a PASS score >=3 (which is in favor of a tumor of aggressive potential) in 2.3% of cases. The anatomical-pathological data showed an adrenocortical carcinoma in 5.8% of the cases with a mean tumor size of 5.75+/- 1.5 cm, a Weiss score >3 in 5.8% of the patients, with a stage I according to the European Network for the Study of Adrenal Tumors (ENSAT) classification in 2.3% and a stage II in 3.4% of cases. The anatomopathological examination of the rest of the operated patients was in favor of a cortisolic adenoma with a mean tumor size of 4.9+/- 0.7 cm with a Weiss score <3.

These histological data guided the surveillance modalities. The follow-up of the operated patients did not show any tumor recurrence, on the other hand, the follow-up of the non-operated patients showed a significant increase in tumor size of more than 20% of the initial size in 2.3% of the patients, thus requiring surgery.

## Discussion

Cross-sectional imaging is frequently used to visualize Als [1]. The prevalence of adrenal incidentaloma (AI) increases with age, with a peak incidence between the fifth and seventh decade, which is similar to our series [2-4]. In Korean patients, AIs were more frequent in men (57%), whereas in our series, AIs were found more in women. During our study period, Als were identified most often on a scanner in 62% of cases requested for digestive signs and were unilateral in 73.25%, which is in concordance with the literature [2-4]. A large number of clinical studies have investigated the characteristics of AIs [3]. It suggests a higher prevalence of left-sided adrenal tumors detected on imaging [5-8] similar to what our results suggest. Song et al. reported a mean AI diameter of 30 mm [3], agreeing with the mean diameter of our series, which was 35.5 mm. However, the incidence of adrenocortical carcinoma becomes higher when the size exceeds 4 cm, more frequent in females with an age between 40 and 50 years, such as in our study [9-11]. All patients with adrenal incidentaloma should undergo biochemical screening for pheochromocytoma because these tumors can be clinically silent [12-15]. Hormonal exploration identified 5.81% of pheochromocytomas vs 1.5-14% of cases found in the literature [16-17], mild cortisol secretion without frank clinical Cushing's syndrome in 19.76% of our patients vs 10% in other series [18-19]. Adrenocortical carcinoma is the least frequent in the AI series [10,18]. ACTH was requested to confirm the degree of braking of the corticotropic axis before surgery [19]. Among patients with adrenal incidentaloma, primary hyperaldosteronism is less common than subclinical hypercortisolism and pheochromocytoma. It represents 1.16% in our series and 3.3% of incidentalomas in other series [12]. Once the diagnosis is established, patient-specific factors guide decisions regarding medical or surgical treatment. Up to 21% of adrenal incidentalomas in patients with a history of or known current primary cancer indicate adrenal metastasis. Cancers that are most likely to spread to the adrenal glands are lung cancer, gastrointestinal cancer, melanoma, and renal-cell carcinoma [19-20]. In our series, we have two cases of adrenal metastasis secondary to bronchial tumors. The management of patients with adrenal incidentalomas is individualized according to the guidelines of the European Society of Endocrinology Clinical Practice [12]. Unilateral adrenalectomy is indicated in cases of pheochromocytoma, malignant tumors, and functional unilateral adenoma [19], which was the case in 19.76% of our series. The indication for surgery depends on the degree of hypercortisolism, age, and comorbidities; otherwise, only monitoring is required [19]. Therapeutic abstention is indicated in cases of benign non-functional adenoma less than 4 cm, which was the case in 41.86% of the cases, in agreement with the data in the literature [6].

Strengths and limitations of the study

We were able to study the different clinical, biological, and radiological parameters in order to establish multiple etiological diagnoses and to better orient the therapeutic management. However, this study was limited by the small sample size because it included only patients from a single university hospital with limited population access. Although the management of adrenal incidentalomas has changed throughout the years our study was conducted, these circumstances have not affected the results of the study in any way or form.

## Conclusions

Adrenal incidentalomas are becoming increasingly common with advances in imaging. Our study focuses on the clinical and hormonal characteristics of patients with AI. We highlight the etiological profile of adrenal incidentalomas in the Moroccan population and the main clinical and paraclinical features that could help clinicians better manage these AIs and optimize their management.

Hormonal exploration is essential in all AIs with the determination of urinary/plasma metanephrines in search of pheochromocytoma and a minute braking test with 1 mg of dexamethasone in search of subclinical hypersecretion of cortisol. The measurement of aldosterone and plasma renin is necessary in case of arterial hypertension and/or hypokalemia. Radiological exploration by CT or MRI of the adrenal gland can reliably distinguish benign lesions. The management of AI is individualized on a case-by-case basis. It can involve abstention, simple surveillance, or surgical treatment.
